# Population Genomics and Inference of *Mycobacterium avium* Complex Clusters in Cystic Fibrosis Care Centers, United States

**DOI:** 10.3201/eid2711.210124

**Published:** 2021-11

**Authors:** Nabeeh A. Hasan, Rebecca M. Davidson, L. Elaine Epperson, Sara M. Kammlade, Sean Beagle, Adrah R. Levin, Vinicius Calado de Moura, Joshua J. Hunkins, Natalia Weakly, Scott D. Sagel, Stacey L. Martiniano, Max Salfinger, Charles L. Daley, Jerry A. Nick, Michael Strong

**Affiliations:** National Jewish Health, Denver, Colorado, USA (N.A. Hasan, R.M. Davidson, L.E. Epperson, S.M. Kammlade, S. Beagle, A.R. Levin, V. Calado de Moura, J.J. Hunkins, N. Weakly, C.L. Daley, J.A. Nick, M. Strong);; University of Colorado Anschutz Medical Campus and Children’s Hospital Colorado, Aurora, Colorado, USA (S.D. Sagel, S.L. Martiniano);; University of South Florida College of Public Health and Morsani College of Medicine, Tampa, Florida, USA (M. Salfinger)

**Keywords:** *Mycobacterium avium* complex, *Mycobacterium intracellulare* subsp. *intracellulare*, *Mycobacterium intracellulare* subsp. *chimaera*, transmission, clusters, whole-genome sequencing, phylogenomics, cystic fibrosis, tuberculosis and other mycobacteria, nontuberculous mycobacteria, bacteria, United States

## Abstract

*Mycobacterium avium* complex (MAC) species constitute most mycobacteria infections in persons with cystic fibrosis (CF) in the United States, but little is known about their genomic diversity or transmission. During 2016–2020, we performed whole-genome sequencing on 364 MAC isolates from 186 persons with CF from 42 cystic fibrosis care centers (CFCCs) across 23 states. We compared isolate genomes to identify instances of shared strains between persons with CF. Among persons with multiple isolates sequenced, 15/56 (27%) had >1 MAC strain type. Genomic comparisons revealed 18 clusters of highly similar isolates; 8 of these clusters had patients who shared CFCCs, which included 27/186 (15%) persons with CF. We provide genomic evidence of highly similar MAC strains shared among patients at the same CFCCs. Polyclonal infections and high genetic similarity between MAC isolates are consistent with multiple modes of acquisition for persons with CF to acquire MAC infections.

Nontuberculous mycobacteria (NTM) are ubiquitous microorganisms found in indoor and outdoor habitats, including water, soil, and dust. NTM can infect susceptible persons, including those with lung diseases such as cystic fibrosis (CF) (*1*). Previous surveys conducted in the United States have found that *Mycobacterium avium* complex (MAC) species are clinically relevant and the most frequently isolated NTM (*2*). MAC consists of 9 slow-growing mycobacterial species (*3*–*6*), of which the 2 most frequently observed are *M. avium* (MAV) and *M. intracellulare*, including its subspecies *intracellulare* (MINT) and subspecies *chimaera* (MCHIM) (*4*). In the United States, most persons with CF and positive NTM cultures (61%) had MAC species infections (*2*,*7*). MAC infections increased by 3% annually during 2010–2014.

MAC pulmonary infections are probably acquired by inhalation of aerosols (*8*), but the sources and modes of transmission of MAC remain unclear. Studies using various molecular genotyping methods have shown MAC isolates from human airway samples to have high genetic similarity to isolates from animals (*8*–*10*), water (*11*,*12*), bathroom faucets (*13*), showerheads (*14*,*15*), pools (*16*), and soil (*17*). Other potential MAC infection sources include fomites, zoonotic sources, and contaminated materials (*10*,*18*). Despite the clinical relevance of MAC and its prevalence among persons with CF, the genomic relationships of MAC isolates and the potential for person-to-person transmission are poorly understood. Whole-genome sequencing (WGS) to analyze the genetic diversity of MAC is aimed at identifying MAC infections that cluster by high bacterial genomic sequence similarity, particularly in susceptible populations such as persons with CF. Unclustered isolates are unrelated and are therefore not implicated in transmission, but clustering between MAC isolates suggests that they are derived from the same source (i.e., shared water, surfaces, or person-to-person transmission). To this end, we analyzed the WGS of NTM isolates voluntarily sent from US CF care centers (CFCCs) during a 4-year period. The goals of this project were to support routine clinical care through high-resolution taxonomic identification, understand the genetic diversity of CF-associated MAC isolates, and identify genetically similar strains among persons with CF for epidemiologic follow-up.

## Materials and Methods

Ethics approval for this work was obtained from the National Jewish Health Institutional Review Board (approval no. HS-3149). As part of Colorado Research and Development Program (https://www.nationaljewishhealth.org/cocfrdp), NTM isolates from US CFCCs were processed and biobanked with the goal of surveillance for genetically similar strains (Table 1). We cultured bacterial samples on Middlebrook 7H11 agar plates (ThermoFisher Scientific, https://www.thermofisher.com) supplemented with 10% oleic acid, albumin, dextrose, catalase growth supplement before subculturing single-colony isolates into Middlebrook 7H9 broth (ThermoFisher Scientific) supplemented with 10% albumin, dextrose, catalase growth supplement and 0.05% Tween 80 (Sigma-Aldrich, https://www.sigmaaldrich.com). We divided these cultures into 1-mL biobanked glycerol stock aliquot replicates that we stored at –20°C. 

**Table 1:** Number of MAC isolates in a study of MAC clusters in cystic fibrosis centers, United States*

Category	MAV	MCHIM	MINT	Total
Patients with 1 isolate, no.	63	33	43	137
Patients with >2 isolates, no.	30	5	23	55
Total patients, no.	93	38	66	186
Total isolates, no.	186	44	134	364

*Some patients have isolates from multiple MAC species. MAC, *Mycobacterium avium* complex; MAV, *M. avium*; MCHIM, *M. intracellulare* subsp. *chimaera*; MINT, *M. intracellulare* subsp. *intracellulare*.

### DNA Extraction and Whole-Genome Sequencing

We extracted NTM DNA as described previously (*19*). We used NexteraXT DNA or DNA FLEX sample preparation (Illumina, https://www.illumina.com) to prepare WGS libraries and sequenced the libraries by using the Illumina MiSeq or HiSeq 2500. WGS data are available at the National Center for Biotechnology Information (BioProject no. PRJNA319839).

### Non-CF Sample Acquisition

To place RDP isolates in context with zoonotic, environmental, and clinical samples from around the world, we included additional MAC isolates with existing WGS in the study. We downloaded 874 MAC genomes from the National Center for Biotechnology Information, including MAV (559 total; 42 environmental, 467 non-CF clinical, and 50 zoonotic), MCHIM (114 total; 3 environmental and 111 non-CF clinical), and MINT (201 total; 4 environmental, 192 non-CF clinical, and 5 zoonotic) from 32 published studies (Appendix 1 Table 1) for subsequent comparisons.

### MAC Species Identification

We trimmed sequence reads of adapters and base calls with quality scores <Q20 by using Skewer (*20*). We then assembled trimmed reads into scaffolds by using Unicycler (*21*). We compared genome assemblies against a collection of reference genomes (Appendix 1 Table 1) to estimate average nucleotide identity (ANI) and assign a species call to each isolate (*22*,*23*). A cutoff ANI of >95% indicated the isolate and reference genome belonged to the same species.

### Phylogenomic Analysis

On the basis of taxonomic assignment, with the highest ANI score >95% for each genome, we mapped trimmed sequence reads to respective reference genomes (e.g., *M. avium* strain H87 [*24*]; *M. intracellulare* subsp. *chimaera* CDC 2015-22-71 [*25*]) by using Bowtie2 (*26*). We identified single-nucleotide polymorphisms (SNPs) as previously described (*27*).

By using the genome coordinates that correspond to the partial *rpo*B region used in clinical diagnostics, we extracted sequences from each MAC isolate. We compared the partial *rpo*B sequences from MAV, MCHIM, and MINT phylogenetically by using neighbor-joining and 250 bootstraps of the observed SNPs in MEGA (*28*).

To evaluate relationships between MAV from US CFCCs and global strains, we assessed the phylogenetic relationships to publicly available genomes from 559 non-CF MAV isolates, including 465 clinical, 42 environmental, and 50 zoonotic isolates from Japan, Germany, Belgium, the United Kingdom, the United States, and 12 other countries (Appendix 1 Table 1). To evaluate relationships between MCHIM from US CFCCs with US and global strains, we assessed the phylogenetic relationships to publicly available genomes from 114 non-CF MCHIM isolates, including 109 clinical and 5 environmental isolates from the United Kingdom, the United States, Switzerland, South Korea, Canada, and South Africa (Appendix 1 Table 1). To evaluate relationships between MINT from US CFCCs with US and global strains, we assessed the phylogenetic relationships to publicly available genomes from 201 non-CF MINT isolates, including 192 clinical, 4 environmental, and 5 zoonotic isolates from China, the United Kingdom, South Korea, and the United States (Appendix 1 Table 1).

### Identifying Genetically Similar Isolate Clusters

**Figure 1:**
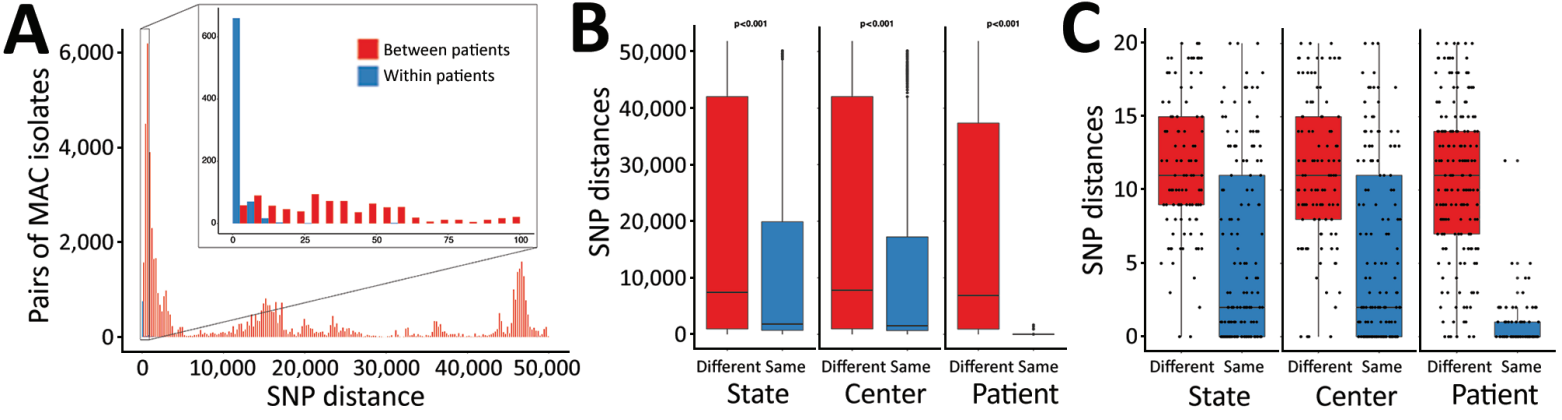
Cluster analysis of MAC in persons with cystic fibrosis to identify recent shared ancestry in a study of MAC clusters in cystic fibrosis centers, United States. A) Pairwise SNP distances of *Mycobacterium avium* and *M. intracellulare* subsp. *chimaera*, and *M. intracellulare* subsp. *intracellulare* isolates from within same patients (blue) and between different patients (red). B) Pairwise SNP distances of all CFCC MAC by state, CFCC, and patient comparisons. Kruskal–Wallis rank-sum test p values for comparing mean differences between categories are specified above each comparison. C) Pairwise SNP distances of CFCC MAC by state, CFCC, and patient comparisons under the clustering threshold. Box and scatterplots in panels B and C show SNPs between isolates at the same versus different states, same versus different CFCC, and same versus different patients. Horizontal lines within boxes indicate medians; top and bottom of boxes indicate 25th and 75th percentiles; error bars indicate the maximum and minimum values observed in the distribution. CFCC, cystic fibrosis care center; MAC, *Mycobacterium avium* complex; SNP, single-nucleotide polymorphism.

To identify a SNP threshold for genetically similar isolates, we examined genomewide SNP distances between pairs of longitudinal isolates from the same person (within-patient isolates) and isolates from different persons (between-patient isolates) in the US CFCC MAC dataset, analogous to methods used previously for *M. abscessus* and MAV (*29**–**33*). The US CFCC MAC dataset included 56 persons with CF who had >2 isolates of the same species: 31 who had >2 MAV isolates, 5 who had >2 MCHIM isolates, and 23 who had >2 MINT isolates. We computed statistical comparisons between MAC groups by using Kruskal–Wallis tests. By using the distributions of within-patient and between-patient genomic SNPs (Figure 1, panel A), we defined a distance of <20 SNPs as the threshold difference for strain definition. We defined isolates found within a patient with a pairwise distance of >20 SNPs as different strains. We notified CFCCs of genetically similar isolates and offered participation in site-specific epidemiologic investigations as part of the ongoing HALT-NTM trial (https://clinicaltrials.gov/ct2/show/NCT04024423) (*34*).

## Results

### Distribution of MAC Species in US Cystic Fibrosis Care Centers

**Figure 2:**
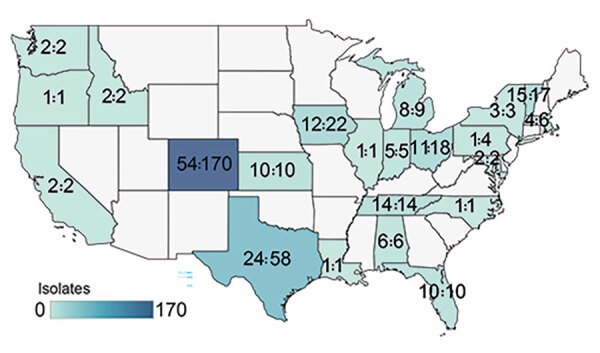
Geographic distribution of 364 *Mycobacterium avium* complex isolates from 186 patients, by cystic fibrosis care center state of origin in study of *M. avium* complex clusters in cystic fibrosis centers, United States. Numbers in each state are the number of patients with cystic fibrosis and total isolates contributed from centers within the state.

**Figure 3:**
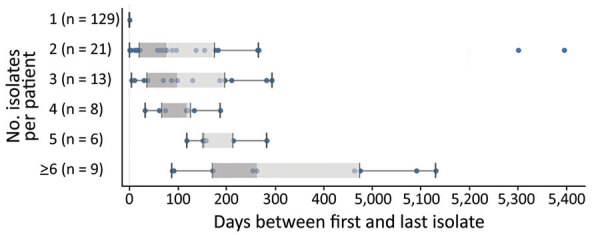
Numbers of isolates per patient and days between the patient’s first and last isolate collected in the isolate cohort in a study of *Mycobacterium avium* complex clusters in cystic fibrosis centers, United States. Vertical lines within boxes indicate medians; top and bottom of boxes indicate 25th and 75th percentiles; error bars indicate the maximum and minimum values observed in the distribution.

We sequenced the genomes of 364 MAC isolates, including 186 MAV (51%), 134 MINT (37%), and 44 MCHIM (12%) (Table 1). More than half (101/186 [54%]) of persons with CF were women or girls (average age 35 years [range 9–88 years]). Isolates were analyzed from a total of 42 CFCCs and 22 states (Figure 2). Two-thirds (129/186 [69%]) of persons with CF had only 1 isolate sequenced, 21 had 2 isolates (21/186 [12%]), and 36 had >3 isolates (36/186 [19%]); collection dates spanned a range of 0 to 1,376 days between the first and last isolate collected (Figure 3). Most (132/186 [71%]) persons with samples analyzed were from 41 CFCCs in 21 states, and the remainder received care at 1 CFCC.

**Figure 4:**
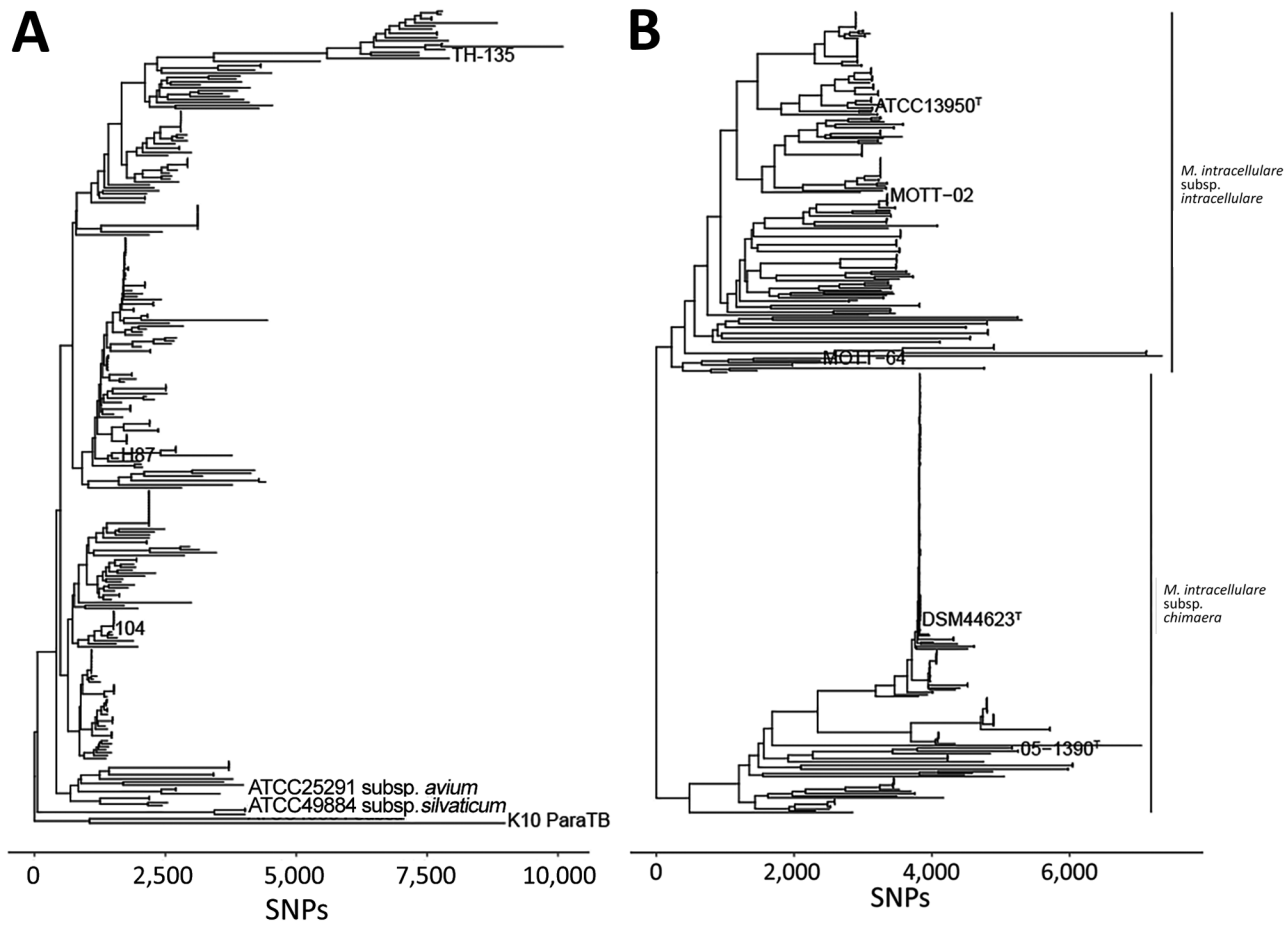
Phylogenetic relationships in a study of *Mycobacterium avium* complex isolates in cystic fibrosis care centers, United States. A) Phylogenetic tree of 207 *M. avium* isolates showing the relationships between *M. avium* cystic fibrosis care center and select non–cystic fibrosis, environmental, and zoonotic isolates. B). Phylogenetic tree of 235 isolates showing the relationships between cystic fibrosis care center and select non–cystic fibrosis *M. intracellulare* subsp. *chimaera* and *M. intracellulare* subsp. *intracellulare* isolates. Former species *M. yongonense* type strain 05–1380^T^ was also included as part of *M. intracellulare* subsp. *chimaera* to reflect current taxonomy. SNP, single-nucleotide polymorphism.

To evaluate taxonomic relationships of closely related taxa, we analyzed isolates from persons with CF, reference genomes for MAV (Figure 4, panel A; Appendix 1 Table 2), and type strains of MINT and MCHIM (Figure 4, panel B; Appendix 1 Table 1). The MAV phylogeny shows that most isolates from persons with CF are *M. avium* subsp. *hominissuis*, except for 1 isolate that was *M. avium* subsp. *avium* (Figure 4, panel A). The *M. intracellulare* phylogeny supports the taxonomy of 2 *M. intracellulare* subspecies, including MCHIM that is distinct from MINT (Figure 4, panel B).

### Polyclonal MAC infections in Persons with Cystic Fibrosis

**Figure 5:**
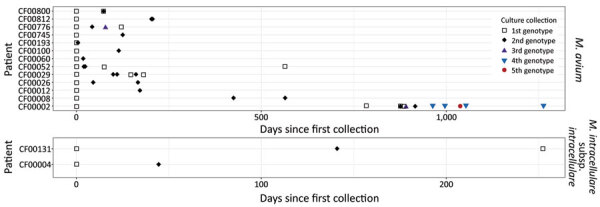
Polyclonal *Mycobacterium avium* complex (MAC) infections in 15 persons with CF in a study of MAC clusters in CF centers, United States. Persons with CF who had >1 MAC isolate were analyzed for the presence of multiple strains within a given MAC species. For *M. avium* (top) and *M. intracellulare* subsp. *intracellulare* (bottom), each row on the y-axis is a person with CF, and the x-axis represents the number of days after the first MAC isolate with whole-genome sequencing was collected. Each point represents a sequenced isolate and the shape represents a unique genotype. The plots do not represent all positive cultures in the patients’ histories, but they illustrate how strains change, alternate, or both over time. In some cases, different strains were isolated on the same day or within a 1-week period. CF, cystic fibrosis.

Among 55 persons with CF who had >2 MAC isolates, we identified 15 (15/55 [27%]) who had multiple strains or species (Figure 5). Nine persons with CF (9/55 [16%]) had isolates from >2 MAC species; 1 (1/55 [2%]) had isolates of MAV, MCHIM, and MINT. Thirteen persons with CF who had MAV (13/30 [43%]) had >2 distinct MAV strains (>500 SNPs apart). Among these 13 persons with CF, we observed an average of 2.3 (range 2–5) different strains/patient; average within-patient diversity was 3,384 SNPs. Two (9%) of 23 persons with CF had 2 different strains of MINT; no persons with MCHIM had multiple strains. In total, 15/55 persons with CF had >1 strain or species, compared with 40/55 (73%) who had the same MAC strain isolated over time.

For the 15 persons with CF who had multiple strains of 1 MAC species, we generated time series plots of longitudinal isolates to visualize changes in strains over time (Figure 5; Appendix 2 Figure 1). The average time from first to last isolate collected was 259 days (range 5–1,262 days). In the case of the shortest interval, patient CF00193 was culture-positive with 2 different strains of MAV collected only 5 days apart. Patients CF00052, CF00060, and CF00193 each had 2 different strains of MAV collected within 30-day windows. Patient CF00002 was culture-positive for 3 different strains of MAV within a single week and had 5 different strains over nearly 3.5 years. In the 2 persons with CF harboring multiple strains of MINT, the second strain was detected 42 days (CF00004) and 138 days (CF00131) after the first isolate collected. Patients CF00029 and CF00776 showed alternating strains over time, suggesting persistent mixed populations of MAV in the airway.

### Assessing Potential Transmission between Persons with Cystic Fibrosis

To evaluate routine molecular surveillance available in most diagnostic laboratories and compare it to the resolution afforded by WGS, we compared the *rpo*B partial sequences of each MAV, MCHIM, and MINT from US persons with CF. For MAV, 100% of patients belonged to 1 of 4 clusters (Appendix 2 Figure 2, panel A) based on analyses using *rpo*B, whereas 97.2% of MCHIM and 95.5% of MINT belonged to 5 clusters (Appendix 2 Figure 2, panel B). This result emphasizes that single-gene amplicon surveillance does not provide the resolution needed for genetic surveillance of NTM MAC species, whereas WGS does provide the necessary resolution.

**Figure 6:**
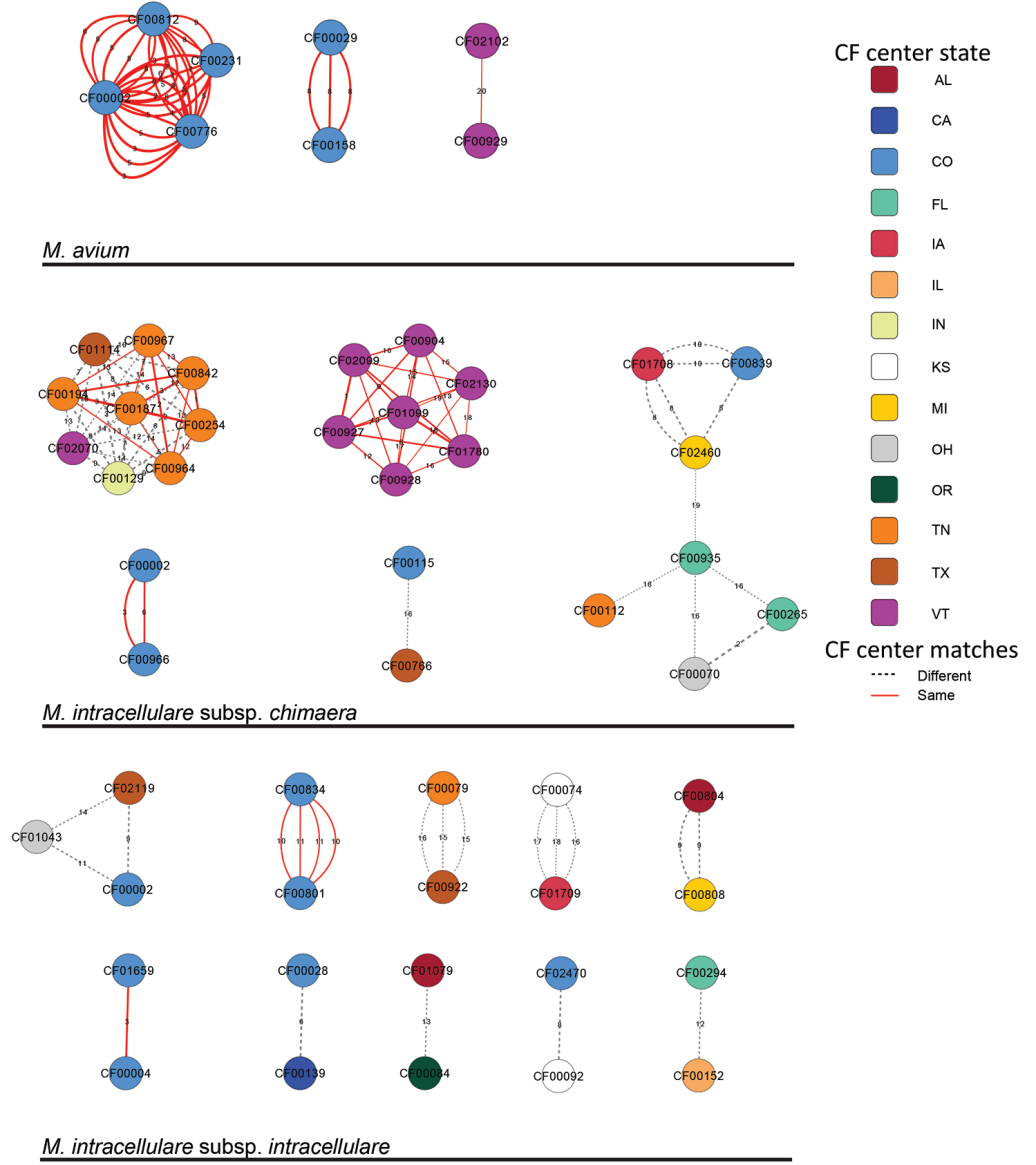
Genetic clusters of *Mycobacterium avium*, *M. intracellulare* subspecies *chimaera*, and *M. intracellulare* subsp. *intracellulare* in persons with CF in a study of *Mycobacterium avium* complex clusters in cystic fibrosis centers, United States. Three clusters *of M. avium*, 5 clusters of *M. intracellulare* subsp. *chimaera*, and 10 clusters of *M. intracellulare* subsp. *intracellulare* were identified. Each node represents a patient with >1 isolate having significant genetic similarity to an isolate in >1 patient. The color of each node represents the state of the submitting CF care center. Each edge represents genetic similarity between the isolates. Connecting edges are colored by matches within a center (red) or between different centers (dashed gray), and edge thickness is weighted from 0 SNPs (thickest) to 20 SNPs (thinnest) and the exact number of SNPs specified. Nodes with multiple connecting edges represent multiple isolates matching between patients. CF, cystic fibrosis; SNP, single-nucleotide polymorphism.

To examine potential transmission of MAC isolates between persons with CF, we identified 20 SNPs as the threshold for recent shared ancestry on the basis of the distribution of SNPs among longitudinal isolates collected over time (Figure 1, panel A). By using this threshold, we identified a total of 18 genetically similar clusters, including 3 MAV, 5 MCHIM, and 10 MINT clusters (Figure 6). Of the 3 MAV clusters, 2 clusters consisting of 6 patients receiving treatment at 1 CFCC, and a third cluster consisting of 2 patients from a second CFCC. Most patients (15/27 [56%]) in 3/5 MCHIM clusters received treatment in the same CFCCs, whereas the remaining isolates in clusters originated from patients attending different CFCCs. Alternatively, a minority (4/21 [19%]) of patients in 2/10 MINT clusters received treatment in the same CFCC, suggesting that MINT may have different transmission routes compared with MAV or MCHIM. Among the entire US CFCC MAC dataset, 8/93 persons with MAV (9%), 15/36 with MCHIM (42%), and 4/66 with MINT (6%) belonged to clusters within the threshold of 20 SNPs and were treated at the same CFCCs, triggering epidemiologic follow-up in the HALT-NTM Trial (*34*). By using a 10-SNP threshold, we identified 2 *M. avium* clusters, 5 *M. chimaera*, and 6 *M. intracellulare* clusters (Appendix Figure 7). Overall, 4 patients included in 2 clusters defined by a 20-SNP threshold are removed when the threshold is reduced to 10 SNPs.

**Figure 7:**
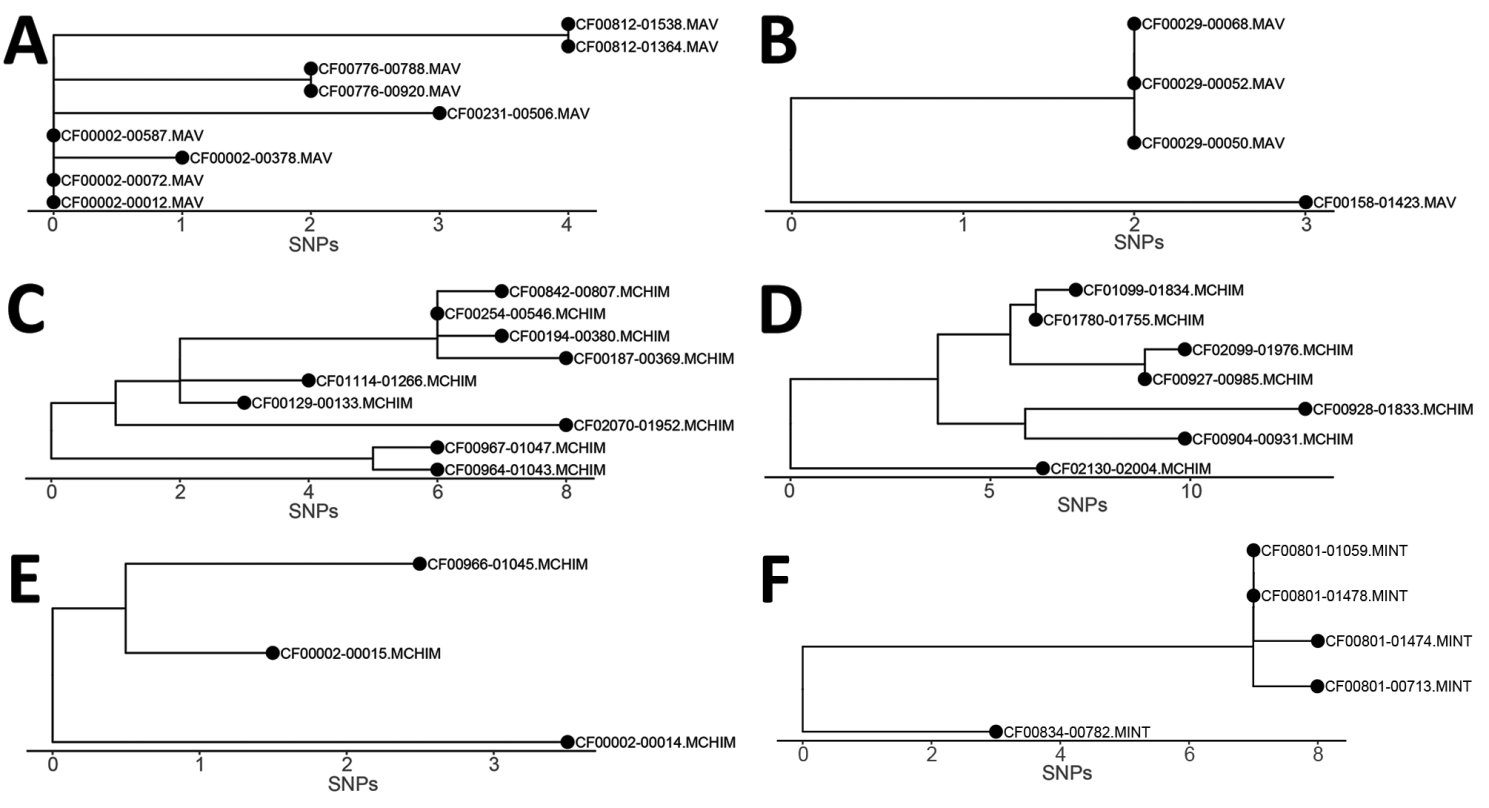
Phylogenetic visualization of *Mycobacterium avium* complex clusters in persons with CF in a study of *M. avium* complex clusters in CF centers, United States. Clusters with >3 isolates were visualized as clades to show the transition of patients’ isolates over time. A) Cluster of 4 persons with MAV. B) Cluster of 2 persons with MAV. C) Cluster of 9 persons with MCHIM). D) Cluster of 7 persons with MCHIM. E) Cluster of 2 persons with MCHIM. F) Cluster of 2 persons with MINT. CF, cystic fibrosis; MAV, *M. avium*; MCHIM, *M. intracellulare* subsp. *chimaera*; MINT, *M. intracellulare* subsp. *intracellulare*; SNP, single-nucleotide polymorphism.

Overall, 27/186 persons with CF (15%) had MAC isolates that were genetically similar and received treatment at the same CFCC. Isolates collected within the same center were more similar than isolates collected from the same state (p = 0.014), whereas the mean SNPs observed between isolates coming from different centers were not significantly different from those coming from different states (Figure 1, panel B). The mean SNP differences observed between nearest-neighboring clustered MAC isolates from the same versus different CFCCs (5.47 vs. 11.21 SNPs; p<0.001) and the same versus different states (5.45 vs. 11.46 SNPs; p<0.001) were both significant (Figure 1, panel C). Only 2 clustered patient pairs (4/186 [2%]) were identified between different centers within a state, suggesting that clustering is more localized to CFCCs than to states.

For isolate clusters that included >3 isolates, we visualized the isolate relationships as phylogenetic clades (Figure 7). The patient with the isolate nearest to the base of each clade is ancestral to all descendants, and therefore is a potential source of transmission between the subsequent patients in the cluster. For example, patient CF00002 was the potential source of 2 separate clusters of MAV and MCHIM. In the MAV cluster (Figure 7, panel A), 4 isolates from patient CF00002 were ancestral to isolates from 3 other patients (CF00231, CF00776, and CF00812). In the MCHIM cluster, 2 isolates from patient CF00002 were ancestral to 1 isolate from patient CF00966 (Figure 7, panel E). Ancestral isolates and hypotheses about the order in which transmission events occurred can similarly be deduced for an additional MAV cluster (Figure 7, panel B), 3 MCHIM clusters (Figure 7, panels C–E), and 1 MINT cluster (Figure 7, panel F).

### MAV

By using a genetic similarity threshold of 20 SNPs, we observed limited instances of genetic similarity between US CFCC MAV isolates from 11 persons with CF and 21 non-CF isolates (Appendix 2 Figure 3). Four persons with CF had genetically similar MAV isolates to an environmental isolate collected from a household dust sample in Germany (Table 2; Appendix 2 Figure 4). Comparisons of US persons with CF MAV isolates to non-US clinical and zoonotic MAV isolates revealed similarities with 17 clinical isolates from patients in 6 countries (Belarus, Canada, Germany, Norway, United Kingdom, and United States), 3 zoonotic isolates from 2 birds (*35*), and 1 from an elephant. Overall, only 11/93 (12%) of persons with CF shared genetically similar isolates with non-CF MAV isolates.

**Table 2:** Persons with CF with genetically similar MAC isolates compared with publicly available non-CF isolates collected from environmental, clinical, and zoonotic sources in a study of MAC clusters in CF centers, United States*

Taxon	No. (%)			
	Environmental	Non-CF clinical	Zoonotic	Total similar CF patients
MAV	4/93 (5)	9/93 (12)	3/93 (3)	11/93 (12)
MCHIM	0/38 (21)	28/38 (61)	0/38 (0)	28/38 (74)
MINT	0/66 (0)	14/66 (24)	3/66 (5)	14/66 (21)
Total				52/186 (28)

*Some patients have isolates that match to an isolate (or isolates) in >1 category. CF, cystic fibrosis; MAC, *Mycobacterium avium* complex; MAV, *M. avium*; MCHIM, *M. intracellulare* subsp. *chimaera*; MINT, *M. intracellulare* subsp. *intracellulare*.

### MCHIM

A total of 30 MCHIM isolates from 28 persons with CF were similar to 37 non-CF isolates (Appendix 2 Figure 3). Matches to US CFCC isolates also include the MCHIM type strain DSM44623^T^, 21 isolates from Oxford Hospital (Oxford, UK), and isolates from patients treated in Canada, Hawaii, and Virginia (Table 2; Appendix 2 Figure 5). US CFCC MCHIM isolates were all genetically different from isolates derived from contaminated heater–cooler units (*36*). No other environmental MCHIM isolates were available for comparisons. In total, 28/38 (74%) persons with CF and MCHIM had genetically similar isolates to non-CF isolates.

### MINT

For MINT, we observed genetic similarities between isolates from 14 persons with CF and 24 non-CF isolates from North America, Europe, and Asia (Appendix 2 Figure 3). Eight MINT isolates were genetically similar to reference isolates, including MINT MOTT-02 (*37*), NCTC-13025 (*38*), and 22 nonpatient isolates from Michigan, Virginia, South Korea, and the United Kingdom (Table 2; Appendix 2 Figure 5). We did not observe similarities between environmental MINT and US CFCC isolates. Comparisons of US CFCC MINT isolates with zoonotic isolates identified similarity with isolates collected from a bird in a California zoo and the other from a penguin in a New York State zoo (*35**,**39*). Overall, 14/66 (21%) persons with CF and MINT had isolates with genetically similar matches to our non-CF isolate sample set.

## Discussion

This study provides evidence of highly similar MAC isolates among persons with CF. However, the isolates from most MAC infections appear to be independently acquired and unclustered. We identified 18 genetically similar isolate clusters involving 54 persons with CF (including 8 patients with MAV, 27 patients with MCHIM, and 21 patients with MINT) within our threshold of recent shared ancestry (<20 SNPs). We further determined that 8 of the identified clusters (8/18 [44%]) included 26 patients that received treatment at the same CFCCs. Person-to-person transmission may have occurred among those persons, and the genetic clusters are undergoing epidemiologic investigation (*34*). Epidemiologic follow-up will help us understand if genetic similarity is related to acquisition through common geography and environments. Most persons with CF (160/186 [86%]) in our study did not share similar strains; thus, we infer that most persons with CF do not transmit strains person-to-person or share acquisition sources of MAC.

In contrast with the clonality observed in *M. abscessus* (*27*,*29*), 27% of patients with MAC cultured multiple strains over time, as has also been observed for *Staphylococcus aureus* infections in persons with CF (*40*). This observation was considerably lower than the proportion of polyclonal MAC infections previously observed in patients with non-CF NTM lung disease (*29*). Although the analysis of single isolates instead of colony sweeps provides the clarity to genetically identify transmission clusters, it may underestimate the diversity of MAC populations present in patient airways. We surmise that MAV isolates found in most US persons with CF probably derive from the independent acquisition (or acquisitions) of strains in the environment. This interpretation is consistent with previously observed instances of genetically matched environmental and patient MAV isolates (*10*,*13*,*14*,*17*,*30*,*41*); however, it does not exclude the hypothesis of person-to-person transmission in persons with CF. Two hypotheses can explain the observations of multiple genotypes and species in persons with CF: patients were originally infected with multiple genotypes of MAC that were selected for during infection and treatment, or patients cleared the original infection and subsequently acquired a new, independent genotype. Our analyses provide evidence for both scenarios (Figure 5), though with limited sample sizes. Further studies of within-patient population diversity with corresponding environmental sampling are needed to address these questions.

Our WGS analysis of 364 MAC isolates, sent from 42 CFCCs in 23 states across the United States as part of a voluntary nationwide surveillance program, enabled us to examine genetic relationships among US isolates. WGS analyses greatly reduced the sizes of MAC clusters identified in US persons with CF compared with *rpo*B sequence information alone, highlighting the value of WGS resolution for epidemiologic follow-up. We also compared CF MAC isolates to isolates from previous studies, including those from environmental, zoonotic, and non-CF clinical sources. In our study, US MAV isolates from persons with CF were mostly distinct from non-CF clinical, environmental, and zoonotic samples from the United States (*30*), Europe (*42*–*44*), and Asia (*12*,*37*,*45*,*46*), although 12% of patients in our study had genetic matches to non-CF isolates. This finding is consistent with observations of human patients and animals harboring identical MAV in Europe (*8*,*9*,*12*,*13*,*42*,*47*,*48*). Similarly, only 21% of persons with CF and MINT had genetically similar isolates to non-CF samples, primarily clinical isolates. Few publicly available environmental isolates of MINT were available for comparison because of the lack of MINT found in water sources (*49*), suggesting that persons with CF likely acquire their MAV and MINT infections from nonhuman reservoirs that were not identified in this study.

In contrast, we observed many matches of MCHIM between CF and non-CF isolates. Indeed, a high proportion of MCHIM from US persons with CF (74% of patients) had matches to non-CF clinical isolates relative to MAV or MINT. One hypothesis to explain clustering of MCHIM is that the observed strains are well-adapted to colonize and persist in a human host. Alternatively, the high genetic similarity of MCHIM isolates may also suggest a lineage that has recently come to prominence in North America. Additional environmental and zoonotic sampling of MAV, MCHIM, and MINT isolates in the United States will be needed to better understand the species-specific risks of MAC infection from these sources.

Our study has some limitations. First, our empirically defined SNP threshold for recent common ancestry is specific for our patient cohort and is limited by the number of persons with CF with >2 isolates and the duration of sampling time frames. Thus, our threshold may miss transmission events that occurred before the sampling period. Second, despite observing genetic matches, epidemiologic links are required to support transmission. Our epidemiologic data were limited to isolate collection date and the CFCC where patients received care. Therefore, our analyses provide hypotheses for traditional epidemiologic follow-up at CFCCs that was beyond the scope of our current project but is being addressed in the HALT-NTM Trial (*34*). Third, the publicly available datasets did not allow a uniform comparison to non-CF clinical, environmental, or zoonotic isolates from each CFCC region for each species. 

Our research study discovered potential instances of transmission between patients and assessed the dynamics of MAC infections in persons with CF. The findings of our US-based surveillance work in persons with CF were not possible without the resolution of WGS and underscore the need for continued epidemiologic follow-up in patients with MAC lung disease, with and without CF, to assist infectious disease control measures and limit the spread of MAC infections where possible.

Appendix 1 Metadata tables for isolates used in population genomics and inference of *Mycobacterium avium* complex clusters in cystic fibrosis centers, United States.

Appendix 2 Additional whole-genome sequencing and single-nucleotide polymorphism phylogenetic analyses used in population genomics and inference of *Mycobacterium avium* complex clusters in cystic fibrosis centers, United States.
