# Supplementary material for: Population Genomics and Inference of Mycobacterium avium Complex Clusters in Cystic Fibrosis Care Centers, United States
Source: Emerg Infect Dis. 2021 Nov;27(11):2836–46. doi: 10.3201/eid2711.210124 (PMC8544995; doi:10.3201/eid2711.210124)
Supplement: Appendix 2 — Additional whole-genome sequencing and single-nucleotide polymorphism phylogenetic analyses used in population genomics and inference of Mycobacterium avium complex clusters in cystic fibrosis centers, United States. [file 21-0124-Techapp-s2.pdf]

Article DOI: https://doi.org/10.3201/eid2711.210124

Population Genomics and Inference of *Mycobacterium avium* Complex Clusters in Cystic Fibrosis Care Centers, United States

Appendix 2

Whole-Genome Sequence and SNP Phylogenetic Analysis

Illumina sequence reads were trimmed using Skewer and assembled into draft genomes using Unicycler (*1*). For each isolate, genome assemblies were compared against a collection of reference nontuberculous mycobacteria (NTM) genomes to assign taxonomic identification based upon the highest estimate the average nucleotide identity (ANI) above 95%.

Based upon the highest ANI score above 95% for each genome, the taxonomic assignment determined the reference genome each isolate's trimmed sequence reads was mapped to (e.g., *M. avium* isolates mapped to *M. avium* strain H87 or *M. intracellulare* subsp. *chimaera* and *M. intracellulare* subsp. *intracellulare* isolates were mapped to *M. intracellulare* subsp. *chimaera* CDC 2015–22–71).

Single nucleotide polymorphisms were called using SAMtools mpileup program (*2*) and were filtered using a custom perl script using the following parameters: SNP quality score of 20, minimum of 4x read depth, the majority of base calls support the variant base, and less than 25% of variant calls occur at the beginning or end of the sequence reads. A multi-fasta sequence alignment was created from concatenated base calls from all isolates. SNP-sites was used to filter positions in the genome in which at least one strain differed from the reference genome, and for which high quality variant and/or reference calls were present for all other isolates (*3*).

To evaluate relationships between MAV from US CF care centers (CFCCs) and global strains, we assessed the phylogenetic relationships to publicly available genomes from 559 non-CF MAV isolates, including 465 clinical, 42 environmental, and 50 zoonotic isolates from Japan

(*4*), Germany (*5*,*6*), Belgium (*7*,*8*), Great Britain (*9*), the U.S (*10–13*). and 12 other countries (Appendix 1 Table 2, https://wwwnc.cdc.gov/EID/article/27/11/21-0124-App1.xlsx).

To evaluate relationships between MCHIM from US CF Care Centers with US and global strains, we assessed the phylogenetic relationships to publicly available genomes from 114 non-CF MCHIM isolates, including 109 clinical, 5 environmental isolates, from the United Kingdom (*14–20*), the U.S (*10*,*12*,*21*), Switzerland (*15*,*22*,*23*), Korea (*24*), Canada (*25*), South Africa (unpublished; Appendix 1 Table 2).

To evaluate relationships between MINT from US CFCCs with US and global strains, we assessed the phylogenetic relationships to publicly available genomes from 201 non-CF MINT isolates, including 192 clinical, 4 environmental, and 5 zoonotic isolates, from China (*26*), Great Britain (*27*), Korea (*10*,*12*,*13*) and the U.S (*28–30*).

To assign genetic relationships within each taxon, we adopted the following hierarchical categories that represent the *least* to *most* genetic similarity as previously described (*31–33*). *Clade* denotes isolates that are monophyletic, *cluster* denotes isolates from different patients that are within a clade and within the SNP threshold of recent common ancestry (*33*). Isolates within a clade with SNP differences exceeding this threshold are considered *unclustered*. To identify clusters between patients, we applied a 20 SNP threshold (Figure 4, https://wwwnc.cdc.gov/EID/article/27/11/21-0124-F4.htm) and a 10 SNP threshold (Appendix 2 Figure 7).

Network visualization of relationships between patients with clustered isolates was performed with Cytoscape 3.8.1 (*34*). Clades of epidemiologically relevant clusters with three or more isolates, collected from two or more patients treated in the same facility were visualized using the MRCA function in the ggtree (*35*).

Treegubbins (*4*) was used to test for the presence of statistically-significant (p = 0.01; 1000 permutations) increases in node density within the *M. avium* and *M. intracellulare* subspecies *chimaera* and *intracellulare* phylogenies. Clades with significant increases in node density were examined for the number of: i) isolates; ii) persons with cystic fibrosis (CF); iii) non-RDP isolates. Clades containing isolates from only one patient were excluded from further designations.

References

1. Wick RR, Judd LM, Gorrie CL, Holt KE. Unicycler: Resolving bacterial genome assemblies from short and long sequencing reads. PLOS Comput Biol. 2017;13:e1005595.

2. Li H, Handsaker B, Wysoker A, Fennell T, Ruan J, Homer N, et al.; 1000 Genome Project Data Processing Subgroup. The Sequence Alignment/Map format and SAMtools. Bioinformatics. 2009;25:2078–9.

3. Page AJ, Taylor B, Delaney AJ, Soares J, Seemann T, Keane JA, et al. *SNP-sites*: rapid efficient extraction of SNPs from multi-FASTA alignments. Microb Genom. 2016;2:e000056.

4. Harris S. TreeGubbins. 2016 [cited yyyy mmm d]. https://github.com/simonrharris/tree_gubbins

5. Yano H, Iwamoto T, Nishiuchi Y, Nakajima C, Starkova DA, Mokrousov I, et al. Population structure and local adaptation of MAC lung disease agent *Mycobacterium avium* subsp. *hominissuis.* Genome Biol Evol. 2017;9:2403–17.

6. Uchiya K, Takahashi H, Yagi T, Moriyama M, Inagaki T, Ichikawa K, et al. Comparative genome analysis of *Mycobacterium avium* revealed genetic diversity in strains that cause pulmonary and disseminated disease. PLoS One. 2013;8:e71831.

7. Sanchini A, Semmler T, Mao L, Kumar N, Dematheis F, Tandon K, et al. A hypervariable genomic island identified in clinical and environmental *Mycobacterium avium* subsp. *hominissuis* isolates from Germany. Int J Med Microbiol. 2016;306:495–503.

8. Lahiri A, Kneisel J, Kloster I, Kamal E, Lewin A. Abundance of *Mycobacterium avium* ssp. *hominissuis* in soil and dust in Germany—implications for the infection route. Lett Appl Microbiol. 2014;59:65–70.

9. Bruffaerts N, Vluggen C, Duytschaever L, Mathys V, Saegerman C, Chapeira O, et al. Genome sequences of four strains of *Mycobacterium avium* subsp. *hominissuis*, isolated from swine and humans, differing in virulence in a murine intranasal infection model. Genome Announc. 2016;4:e00533–16.

10. Quan TP, Bawa Z, Foster D, Walker T, Del Ojo Elias C, Rathod P, et al.; MMM Informatics Group. Evaluation of whole-genome sequencing for mycobacterial species identification and drug susceptibility testing in a clinical setting: a large-scale prospective assessment of performance against line probe assays and phenotyping. J Clin Microbiol. 2018;56:e01480–17.

11. Bryant JM, Thibault VC, Smith DG, McLuckie J, Heron I, Sevilla IA, et al. Phylogenomic exploration of the relationships between strains of *Mycobacterium avium* subspecies *paratuberculosis.* BMC Genomics. 2016;17:79.

12. Chand M, Lamagni T, Kranzer K, Hedge J, Moore G, Parks S, et al. Insidious risk of severe *Mycobacterium chimaera* infection in cardiac surgery patients. Clin Infect Dis. 2017;64:335–42.

13. Pankhurst LJ, Del Ojo Elias C, Votintseva AA, Walker TM, Cole K, Davies J, et al.; COMPASS-TB Study Group. Rapid, comprehensive, and affordable mycobacterial diagnosis with whole-genome sequencing: a prospective study. Lancet Respir Med. 2016;4:49–58.

14. Lande L, Alexander DC, Wallace RJ Jr, Kwait R, Iakhiaeva E, Williams M, et al. *Mycobacterium avium* in community and household water, suburban Philadelphia, Pennsylvania, USA, 2010–2012. Emerg Infect Dis. 2019;25:473–81.

15. Operario DJ, Pholwat S, Koeppel AF, Prorock A, Bao Y, Sol-Church K, et al. *Mycobacterium avium* complex diversity within lung disease, as revealed by whole-genome sequencing. Am J Respir Crit Care Med. 2019;200:393–6.

16. Matern WM, Bader JS, Karakousis PC. Genome analysis of *Mycobacterium avium* subspecies *hominissuis* strain 109. Sci Data. 2018;5:180277.

17. Zhao X, Epperson LE, Hasan NA, Honda JR, Chan ED, Strong M, et al. Complete genome sequence of *Mycobacterium avium* subsp. *hominissuis* strain H87 isolated from an indoor water sample. Genome Announc. 2017;5:e00189–17.

18. Horan KL, Freeman R, Weigel K, Semret M, Pfaller S, Covert TC, et al. Isolation of the genome sequence strain *Mycobacterium avium* 104 from multiple patients over a 17-year period. J Clin Microbiol. 2006;44:783–9.

19. Pfeiffer W, Braun J, Burchell J, Witte CL, Rideout BA. Whole-genome analysis of mycobacteria from birds at the San Diego Zoo. PLoS One. 2017;12:e0173464.

20. Li L, Bannantine JP, Zhang Q, Amonsin A, May BJ, Alt D, et al. The complete genome sequence of *Mycobacterium avium* subspecies *paratuberculosis.* Proc Natl Acad Sci U S A. 2005;102:12344–9.

21. Mac Aogáin M, Roycroft E, Raftery P, Mok S, Fitzgibbon M, Rogers TR. Draft genome sequences of three *Mycobacterium chimaera* respiratory isolates. Genome Announc. 2015;3:e01409–15.

22. Hasan NA, Honda JR, Davidson RM, Epperson LE, Bankowski MJ, Chan ED, et al. Complete genome sequence of *Mycobacterium chimaera* strain AH16. Genome Announc. 2016;4:e01276–16.

23. Hasan NA, Lawsin A, Perry KA, Alyanak E, Toney NC, Malecha A, et al. Complete genome sequence of *Mycobacterium chimaera* strain CDC 2015–22–71. Genome Announc. 2017;5:e00693–17.

24. van Ingen J, Kohl TA, Kranzer K, Hasse B, Keller PM, Katarzyna Szafrańska A, et al. Global outbreak of severe *Mycobacterium chimaera* disease after cardiac surgery: a molecular epidemiological study. Lancet Infect Dis. 2017;17:1033–41.

25. Kim BJ, Kim BR, Lee SY, Seok SH, Kook YH, Kim BJ. Whole-genome sequence of a novel species, *Mycobacterium yongonense* DSM 45126T. Genome Announc. 2013;1:e00604–13.

26. Hasan NA, Warren RL, Epperson LE, Malecha A, Alexander DC, Turenne CY, et al. Complete genome sequence of *Mycobacterium chimaera* SJ42, a nonoutbreak strain from an immunocompromised patient with pulmonary disease. Genome Announc. 2017;5:e00963–17.

27. Zhang X, Lin J, Feng Y, Wang X, McNally A, Zong Z. Identification of *Mycobacterium chimaera* in heater-cooler units in China. Sci Rep. 2018;8:7843.

28. Kim B-J, Choi B-S, Lim J-S, Choi I-Y, Kook Y-H, Kim B-J. Complete genome sequence of *Mycobacterium intracellulare* clinical strain MOTT-64, belonging to the INT1 genotype. J Bacteriol. 2012;194:3268.

29. Kim B-J, Choi B-S, Lim J-S, Choi I-Y, Lee J-H, Chun J, et al. Complete genome sequence of *Mycobacterium intracellulare* clinical strain MOTT-02. J Bacteriol. 2012;194:2771.

30. Kim B-J, Choi B-S, Lim J-S, Choi I-Y, Lee J-H, Chun J, et al. Complete genome sequence of *Mycobacterium intracellulare* strain ATCC 13950(T). J Bacteriol. 2012;194:2750.

31. Hasan NA, Epperson LE, Lawsin A, Rodger RR, Perkins KM, Halpin AL, et al. Genomic analysis of cardiac surgery–associated *Mycobacterium chimaera* infections, United States. Emerg Infect Dis. 2019;25:559–63.

32. Perkins KM, Lawsin A, Hasan NA, Strong M, Halpin AL, Rodger RR, et al. Notes from the field: *Mycobacterium chimaera* contamination of heater-cooler devices used in cardiac surgery—United States. MMWR Morb Mortal Wkly Rep. 2016;65:1117–8.

33. Davidson RM, Hasan NA, Epperson LE, Benoit JB, Kammlade SM, Levin AR, et al. Population genomics of *Mycobacterium abscessus* from United States cystic fibrosis care centers. Ann Am Thorac Soc. 2021 Apr 15 [Epub ahead of print].

34. Bryant JM, Grogono DM, Rodriguez-Rincon D, Everall I, Brown KP, Moreno P, et al. Emergence and spread of a human-transmissible multidrug-resistant nontuberculous mycobacterium. Science. 2016;354:751–7.

35. Shannon P, Markiel A, Ozier O, Baliga NS, Wang JT, Ramage D, et al. Cytoscape: a software environment for integrated models of biomolecular interaction networks. Genome Res. 2003;13:2498–504.

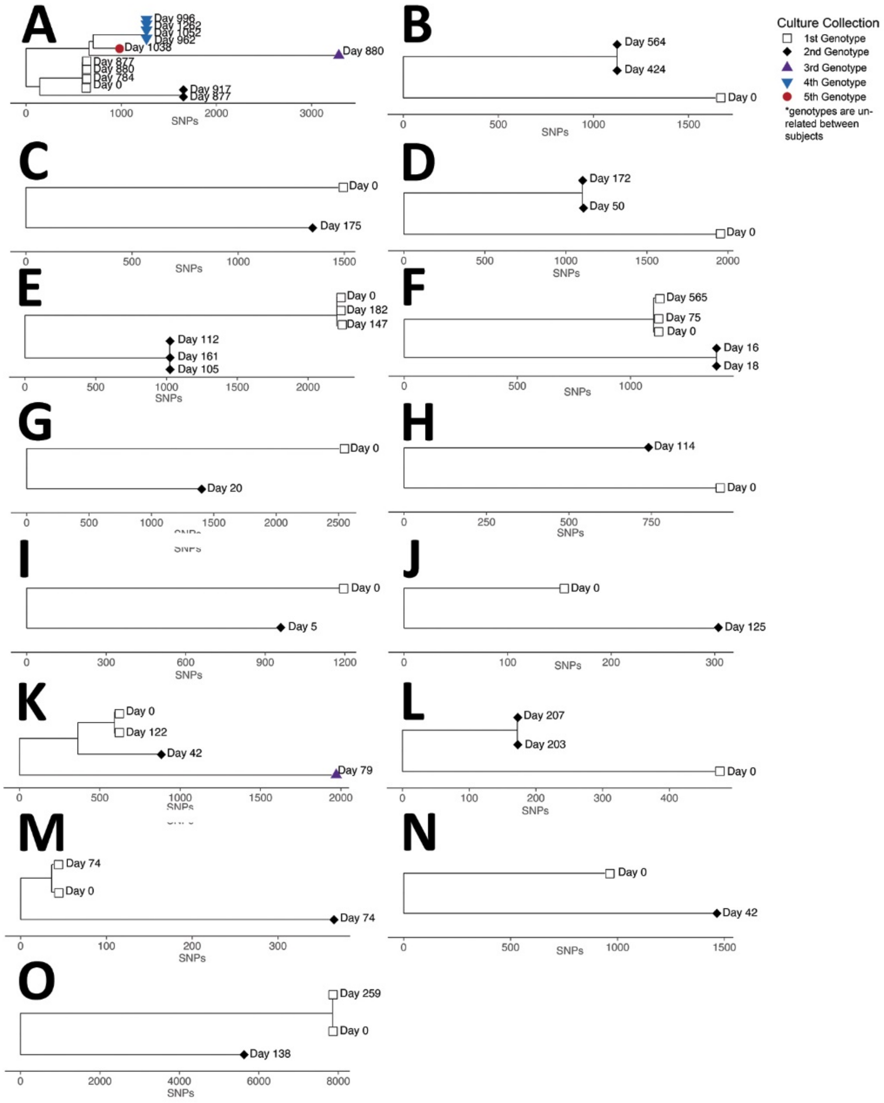

Appendix 2 Figure 1. Phylogenetic visualization of multiple *Mycobacterium avium* and *Mycobacterium intracellulare* subsp. *intracellulare* genotypes found in patients with two or more isolates sequenced. A. Five *M. avium* (MAV) genotypes in 12 isolates collected from patient CF00002. B. Two MAV genotypes in three isolates collected from patient CF00008. C. Two MAV genotypes in two isolates collected from patient CF00012. D. Two MAV genotypes in three isolates collected from patient CF00026. E. Two MAV genotypes in six isolates collected from patient CF00029. F. Two MAV genotypes in five isolates collected from patient CF00052. G. Two MAV genotypes in two isolates collected from patient CF00060. H. Two MAV genotypes in two isolates collected from patient CF00100. I. Two MAV genotypes in two isolates collected from patient CF00193. J. Two MAV genotypes in two isolates collected from patient CF00745. K. Three *M.* MAV genotypes in four isolates collected from patient CF00776. L. Two MAV genotypes in three isolates collected from patient CF00812. M. Two MAV genotypes in three isolates collected from patient CF00880. N. Two *M. intracellulare* subsp. *intracellulare* (MINT) genotypes in two isolates collected from patient CF00004. O. Two MINT genotypes in three isolates collected from patient CF00131.

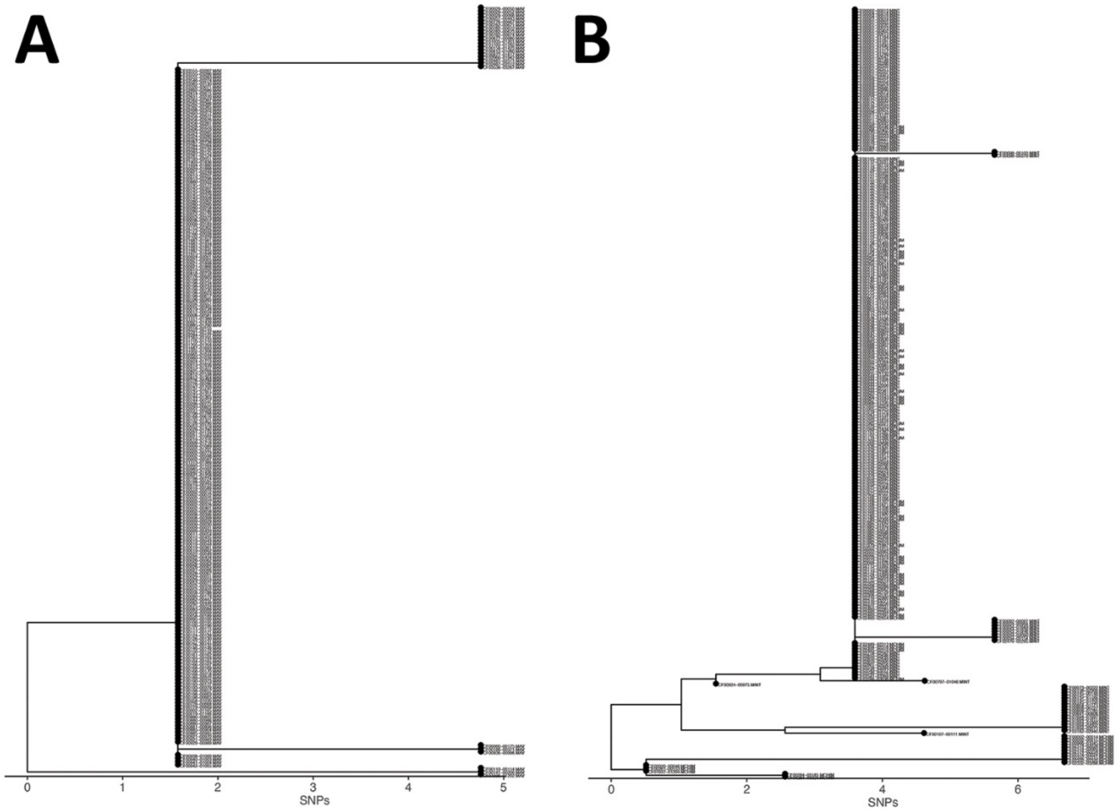

Appendix 2 Figure 2. Phylogenetic clustering of *rpo*B sequences from US CF MAC isolates. A. Phylogeny of 186 *M. avium* (MAV) isolates from 93 patients. B. Phylogeny of 44 *M. intracellulare* subsp. *chimaera* (MCHIM) and 134 *M. intracellulare* subsp. *intracellulare* (MINT) isolates' *rpo*B sequences. *rpo*B from each submitted isolate were analyzed using 250 bootstrap replicates of the observed SNPs and compared to identify phylogenetic clusters.

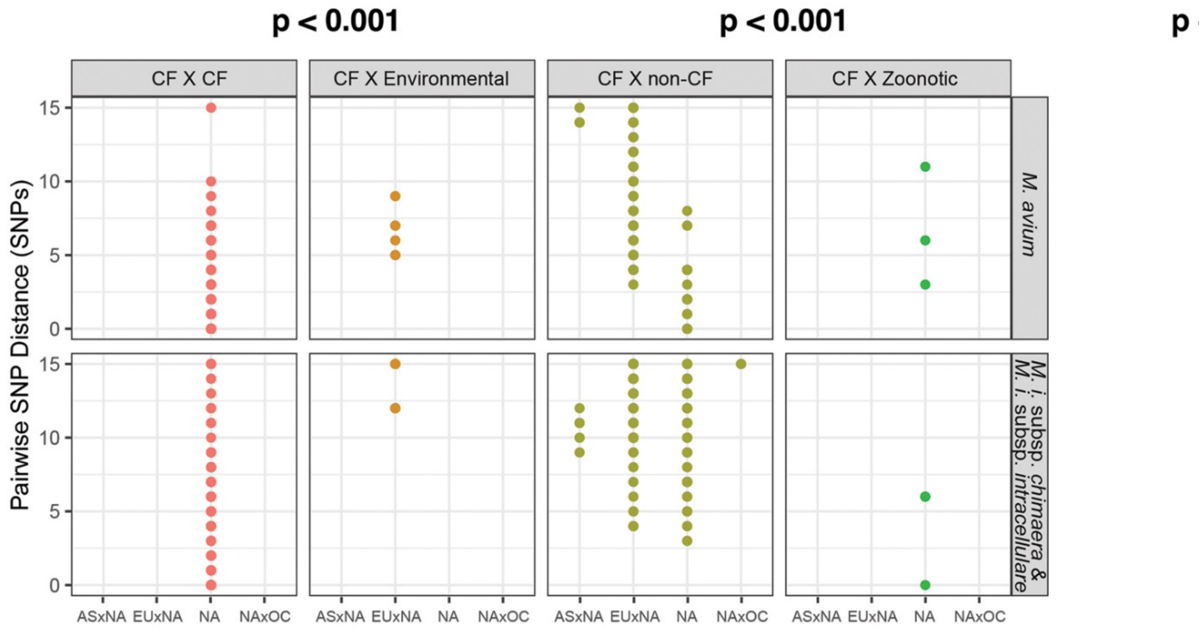

Appendix 2 Figure 3. Matches between US CF Care Center isolates from US persons with CF and environmental, non-CF clinical and zoonotic isolates from around the world. Each row represents the categorical comparisons for *M. avium* (MAV) or *M. intracellulare* subsp. *chimaera* (MCHIM) and *M. intracellulare* subsp*. intracellulare* (MINT). Comparisons between isolates of different continents were arranged along the x-axis are abbreviated for the continents of origin (i.e., Asia: AS; Europe: EA, North America: NA, Oceania: OC).

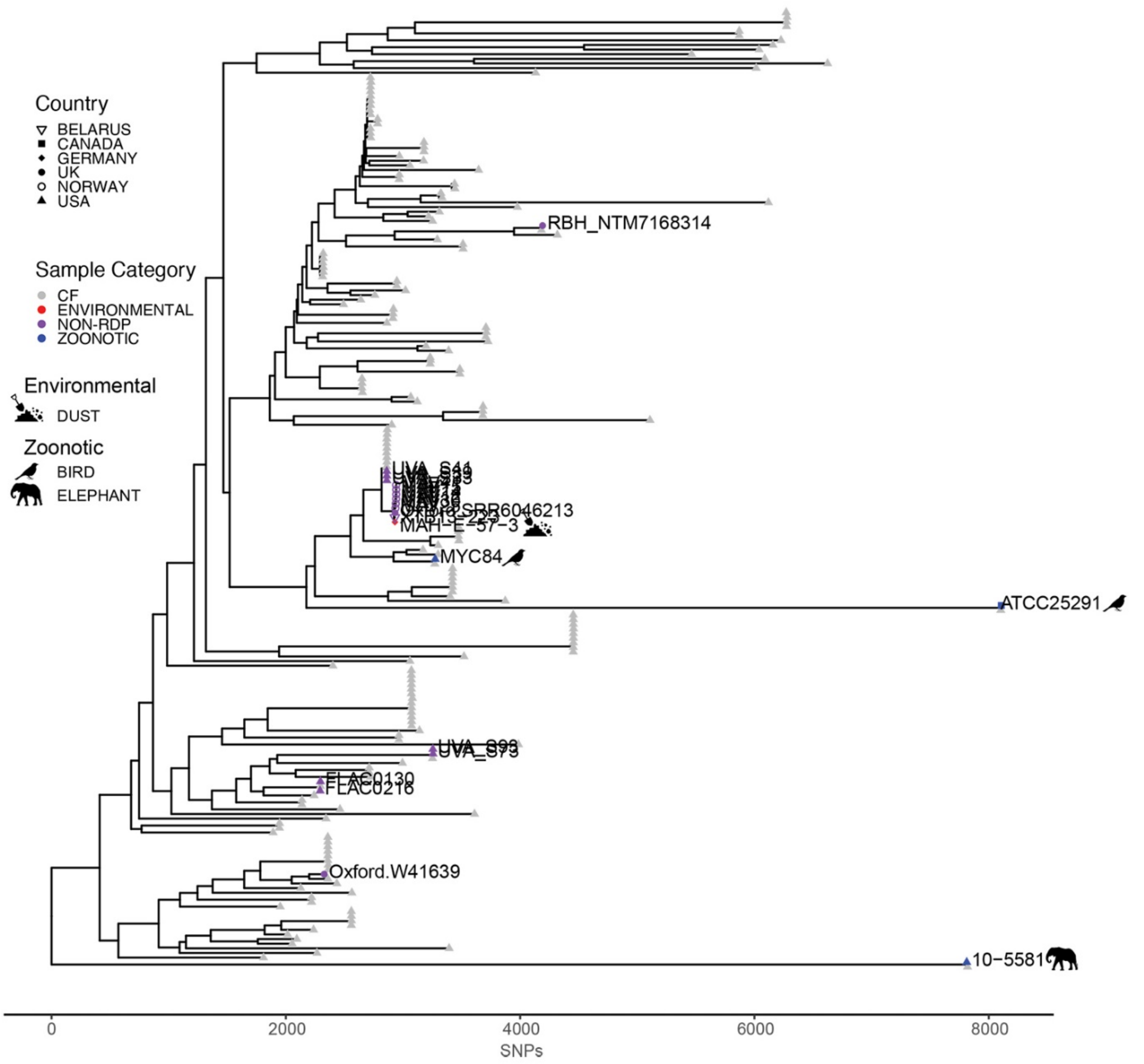

Appendix 2 Figure 4. Phylogenetic Visualization of *M. avium* isolates from US persons with CF and matches to non-CF, Environmental and Zoonotic isolates. Phylogenetic relationships between isolates from patients with CF (gray), environmental (red), non-CF (purple), and zoonotic (blue) isolates from Belarus (downward triangle), Canada (square), Germany (diamond), UK (solid circle), Norway (empty circle) and the USA (triangle) were visualized. Similar environmental isolate was dust-derived (dust symbol) and zoonotic isolates were bird-derived (bird symbol) and elephant-derived (elephant symbol). The scale bar along the x-axis represents the number of single nucleotide polymorphisms (SNPs).

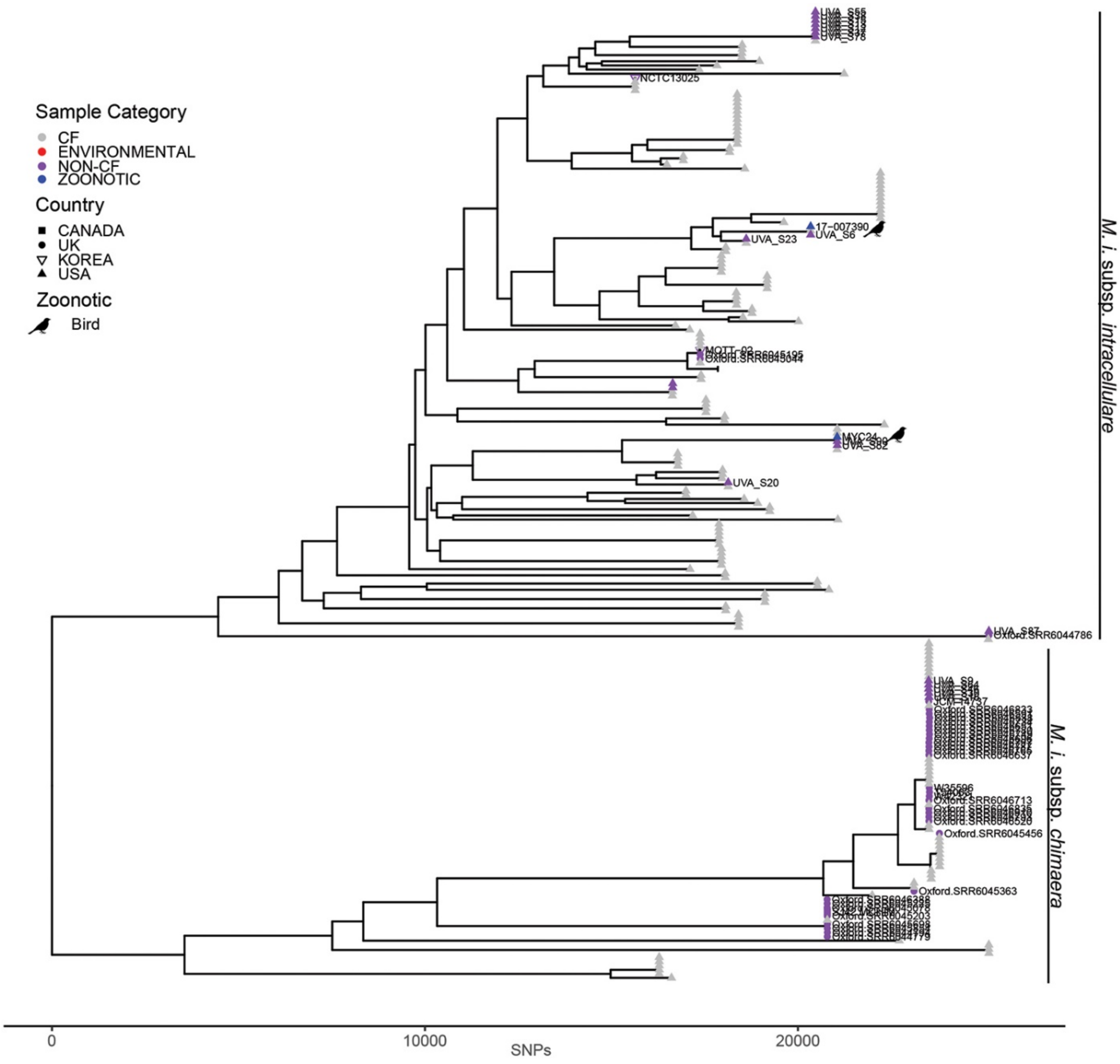

Appendix 2 Figure 5. Phylogenetic Visualization of *Mycobacterium intracellulare subspecies chimaera* and *intracellulare* isolates from US persons with CF and matches to non-CF, Environmental and Zoonotic isolates. Phylogenetic relationships between isolates from persons with CF (gray), environmental (red), non-CF (purple), and zoonotic (blue) isolates from Canada (square), UK (circle), Korea (downward triangle) and the USA (triangle) were visualized. Similar zoonotic isolates were all bird-derived (bird symbol). The scale bar along the x-axis represents the number of single nucleotide polymorphisms (SNPs).

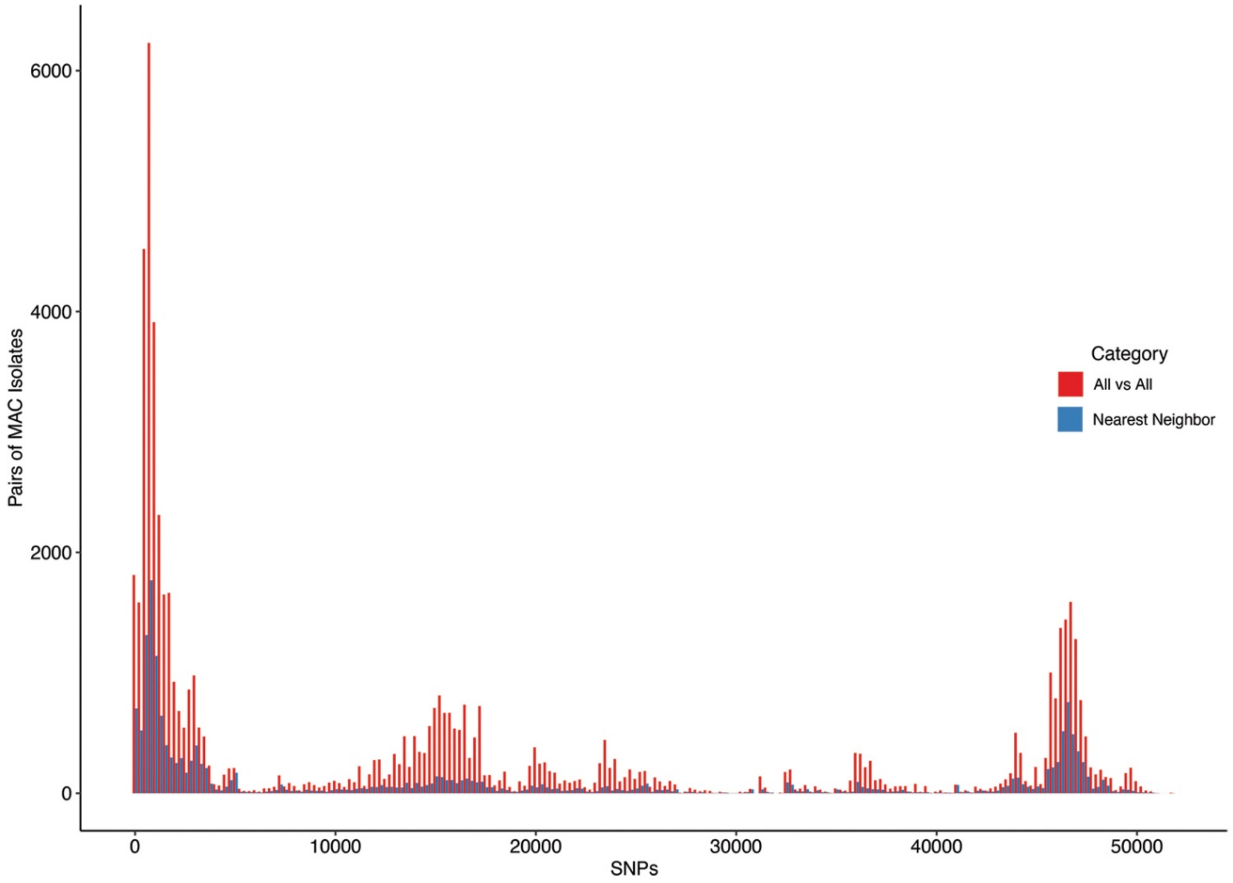

Appendix 2 Figure 6. Pairwise SNP distances of All vs All compared to Nearest Neighbors in MAC isolates infecting US persons with cystic fibrosis. The pairs of isolates (y-axis) having observed SNPs (x-axis) between all *M. avium* (MAV) and *M. intracellulare* subsp. *chimaera* (MCHIM), and *M. intracellulare* subsp. *intracellulare* (MINT) isolates from within the same patient and between different patients (All vs All; red) are compared to the nearest neighbor pair of isolates (blue). with the lowest SNPs observed between all possible pairings of within same patient and between patients is shown. The nearest-neighbor is the combination of two isolates with the lowest pairwise SNP difference observed between each possible pair of patients.

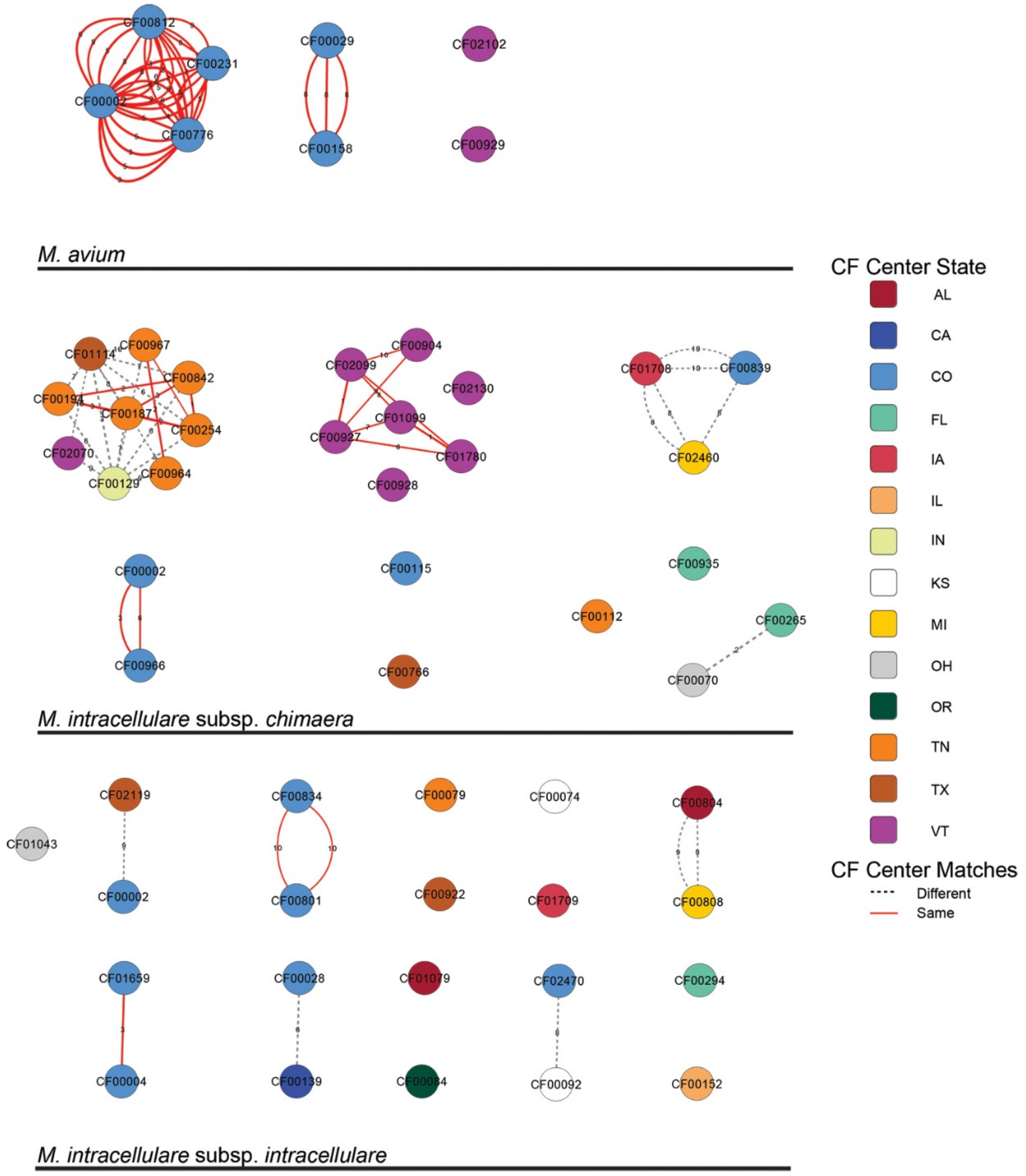

Appendix 2 Figure 7. Genetic clusters of *Mycobacterium avium*, *Mycobacterium intracellulare* subspecies *chimaera* and *intracellulare* in US persons with cystic fibrosis defined by a threshold of 10 SNPs or fewer between patients. Three clusters *of M. avium*, five clusters of *M. intracellulare* subsp. *chimaera* and 10 clusters of *M. intracellulare* subsp. *intracellulare* were identified. Each node represents a patient with at least one isolate having significant genetic similarity to an isolate in one or more patient(s). The color of each node represents the state of the submitting CF Care Center. Each edge represents genetic similarity between the isolates. Connecting edges are colored by matches within a CF Center (red) or between different CF Centers (dashed gray) and edge thickness is weighted from 0 SNPs (thickest) to 10 SNPs (thinnest) and the exact number of SNPs specified. Nodes with multiple connecting edges represent multiple isolates matching between the patients.
